# The Roles of the 5′ and 3′ Untranslated Regions in Human Astrovirus Replication

**DOI:** 10.3390/v15061402

**Published:** 2023-06-20

**Authors:** Nicole Wildi, Torsten Seuberlich

**Affiliations:** 1 Division of Neurological Sciences, Vetsuisse Faculty, University of Bern, 3012 Bern, Switzerland; nicole.wildi@unibe.ch; 2 Graduate School for Cellular and Biomedical Sciences, University of Bern, 3012 Bern, Switzerland

**Keywords:** astrovirus, replication, reverse genetics, untranslated region, translation

## Abstract

Astroviruses are small nonenveloped single-stranded RNA viruses with a positive sense genome. They are known to cause gastrointestinal disease in a broad spectrum of species. Although astroviruses are distributed worldwide, a gap in knowledge of their biology and disease pathogenesis persists. Many positive-sense single-stranded RNA viruses show conserved and functionally important structures in their 5′ and 3′ untranslated regions (UTRs). However, not much is known about the role of the 5′ and 3′ UTRs in the viral replication of HAstV-1. We analyzed the UTRs of HAstV-1 for secondary RNA structures and mutated them, resulting in partial or total UTR deletion. We used a reverse genetic system to study the production of infectious viral particles and to quantify protein expression in the 5′ and 3′ UTR mutants, and we established an HAstV-1 replicon system containing two reporter cassettes in open reading frames 1a and 2, respectively. Our data show that 3′ UTR deletions almost completely abolished viral protein expression and that 5′ UTR deletions led to a reduction in infectious virus particles in infection experiments. This indicates that the presence of the UTRs is essential for the life cycle of HAstV-1 and opens avenues for further research.

## 1. Introduction

Astroviruses are distributed worldwide and infect a broad spectrum of host species, including mammals, birds, fish, and reptiles [1,2]. In humans, astroviral infection is associated with gastrointestinal disease but may also affect the nervous system, causing fatal encephalitis [3,4,5,6,7,8,9]. The classical human astroviruses (HAstVs) are major causes of gastroenteritis in children [2,10]. These classical strains are genetically related and belong to the same virus species, *Mamastrovirus 1*, which has eight serotypes (HAstV-1–8) [11]. Other HAstVs, such as those belonging to the VA/HMO and MLB clades, differ genetically and are related to respiratory, central nervous system, and disseminated infections [12,13,14,15,16,17,18,19,20,21].

Astroviruses are positive-sense single-stranded RNA viruses. Their genomes contain three to four open reading frames (ORFs), with ORF1a encoding nonstructural proteins; ORF1b, connected to ORF1a through leaky scanning, encoding an RNA-dependent RNA polymerase (RdRp); and ORF2 encoding capsid proteins [22]. The fourth ORF, called ORFX, is found in some astroviruses, including the classical HAstV strains, and encodes a viroporin-like protein [23]. The viral genomes contain untranslated regions (UTRs) at the 5′ end of ORF1a and the 3′ end of ORF2. A viral protein (VPg) is linked to the viral RNA at the 5′ terminus, and the 3′ terminus is polyadenylated [24]. The translation of the genomic viral RNA leads to the synthesis of two polyproteins (nsp1a and nsp1ab). The precursor capsid protein is translated from a subgenomic RNA, which is transcribed from the antigenomic viral RNA using a subgenomic promoter [25,26].

The examination of the UTRs of other positive-sense single-stranded RNA viruses has revealed important structures that are essential for the initiation of protein translation and for viral RNA replication [27,28,29,30]. For instance, analysis of the large internal ribosomal entry site elements in the 5′ UTRs of members of the family *Picornaviridae* revealed viral protein synthesis initiation and RNA replication functions [31,32,33]. These mechanisms have been investigated in detail for poliovirus 1 (*Enterovirus C*, family *Picornaviridae*) [34]. The 3′ UTRs of other enteroviruses (family *Picornaviridae*) have also been shown to be involved in RNA replication and viral viability [35]. The 3′ UTR of the Norwalk virus (family *Caliciviridae*) has been suggested to be involved in the expression of the VP1 capsid protein [36]. In members of the families *Caliciviridae* and *Picornaviridae*, several cellular proteins are known to interact with viral RNA and to form RNP complexes [34,37,38,39,40,41,42,43,44,45]. However, in astroviruses, only little is known about the structure and function of the UTRs. In the HAstV-8, it has been shown that a polypyrimidine tract binding (PTB) protein is cross-linked to the 3′ UTR [46,47], as it is the case for caliciviruses [28,39]. Furthermore, for classical HAstVs, in silico analysis showed putative protein binding sites for PTB at the 5′ and 3′ UTRs [47]. On the other hand, a highly conserved stem-loop (SL) structure at the ORF2–3′ UTR junction, termed the stem-loop 2 motif (s2m), is present in human and animal astroviruses, members of the family *Coronaviridae*, and the equine rhinitis B virus (*Picornaviridae: Erbovirus*) [48]. Remodeling of the s2m using antisense oligonucleotides resulted in reduced replication in a chimeric severe acute respiratory syndrome coronavirus 2 (SARS-CoV-2)/HAstV-1 replicon [49]. In a recent study, the s2m of HAstV-1 was partially deleted. After serial passaging of the virus, adaptive changes were observed, i.e., a duplication of the remaining 3′ UTR sequences [50]. All these findings underpin that the s2m and other UTR sequences in RNA viruses have important functions.

In this study, we sought to look at the roles of UTRs in the HAstV-1 life cycle. Using reverse genetics and a dual-reporter replicon system, we investigated the involvement of UTRs in viral protein translation and the production of infectious virus particles.

## 2. Materials and Methods

### 2.1. Prediction of Secondary RNA Structures

To assess the role of UTRs in viral replication, we first analyzed the sequence homology of the UTRs of classical HAstV strains for which complete genomes were available in NCBI GenBank. Then, the structures of the 5′ UTR (first 90 nt) and the 3′ UTR (last 104 nt) of the HAstV-1 genome were predicted using the mfold [51] (http://www.unafold.org/mfold/applications/rna-folding-form.php, accessed: 3 February 2021) and RNAfold [52,53] (http://rna.tbi.univie.ac.at/, accessed: 3 February 2021) programs using the default setting of 37 °C. The models generated by the programs formed the basis for the design and construction of UTR deletion mutants in an established HAstV-1 reverse genetics system [54].

### 2.2. Construction of HAstV-1 Mutants and Replicons

**Figure 1:**
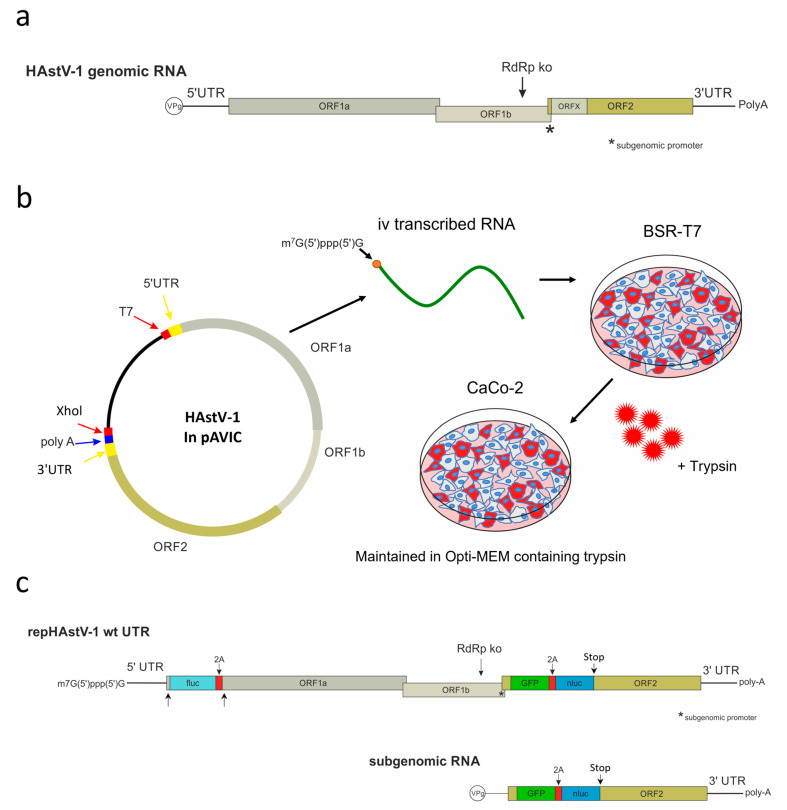
Schematics of the human astrovirus type 1 (HAstV-1) genome organization, showing the RNA-dependent RNA polymerase knockout (RdRp ko) site used to generate a replication-incompetent control mutant from the wild type (wt) (**a**) and the HAstV-1 virus rescue system (**b**). (**c**) Schematics of the HAstV-1 dual-reporter replicons. Two nonlabeled arrows pointing to regions in the ORF1a where the first 45 nt of the ORF1a are placed twice. iv, in vitro; UTR, untranslated region; ORF, open reading frame; VPg, viral protein linked to the genome; GFP, green fluorescent protein; nluc, nanoluciferase; fluc, firefly luciferase.

The HAstV-1 clone in pAVIC [54] was used as a template for the creation of HAstV-1 constructs with 5′ and 3′ UTR deletions (GenBank accession number OR130732). All cloning steps were performed using the In-Fusion^®^ HD cloning kit (Takara Bio, Mountain View, CA, USA) according to the manufacturer’s instructions. The primers used to mutate the UTRs and insert the reporter genes are listed in Table S1. To create a replication-deficient control construct (HAstV-1 RdRp knockout (ko)), we introduced a mutation of two nucleotides in ORF1b (r.3905a > g and r.3906c > g), leading to an amino acid change in the C-motif of the RdRp from aspartic acid to glycine (Figure 1a). This motif is known to be crucial for RdRp activity in other positive-strand RNA viruses [55].

To quantify the protein expression in ORF1a and ORF2, we used a replicon system. As the nonstructural and capsid proteins are translated from genomic and subgenomic RNA, respectively (Figure 1c), we designed dual-reporter replicon for the separate evaluation of protein translation from both RNA types. We inserted a green fluorescent protein (GFP)/nanoluciferase (nluc)-encoding cassette in ORF2 between nt position 4426 and 4427 of the wild-type HAstV genome (OR130732), which corresponds to nt positions 99/100 of ORF2. After the GFP/nluc cassette, a stop codon was inserted to prevent translation of the capsid protein. Enhanced GFP and nluc (pNL1.1.CMV vector; Promega, Madison, WI, USA) were interconnected by a GSG linker sequence and a 2A self-cleaving peptide derived from porcine teschovirus [56]. The cassette was synthesized commercially (Eurofins Genomics, Ebersberg, Germany). In ORF 1a, we inserted a firefly luciferase (fluc) coding sequence between nt position 130 and 131 of the wild-type HAstV-1 genome (OR130732). It was followed by 2A cleavage site and the first 45 nt of the ORF1a again to assure generation of the authentic N-terminus of the nonstructural precursor proteins nsp1a and nsp1ab. This implies that the first 45 nt of ORF1a was kept upstream of fluc, but it was also present downstream of the first 2A cleavage site. The fluc sequence was obtained from the pGL2 plasmid (Promega, Madison, WI, USA). These reporter cassettes were inserted in the wild-type (wt) HAstV-1, the RdRp ko mutant, and the UTR mutants. To confirm sequence correctness, all constructs were sequenced entirely using the BigDye™ Terminator v. 3.1 cycle sequencing kit (Applied Biosystems, Waltham, MA, USA) on a 3730 DNA analyzer (Applied Biosystems, Waltham, MA, USA).

The GFP signal was only used as a qualitative visual readout of RNA replication and protein translation during the development of the replicons. Afterward, all quantification was performed by measuring the luciferase activities.

### 2.3. Cell Culture

Baby hamster kidney 21 cells that constitutively express the T7 RNA polymerase (BSR-T7) [57]) and human epithelial colorectal adenocarcinoma (CaCo-2) cells were maintained in DMEM, high glucose, and pyruvate (Gibco, Carlsbad, CA, USA) supplemented with 10% fetal calf serum and 1% penicillin/streptomycin at 37 °C. The cells were seeded and transfected or infected at 80–90% confluency.

### 2.4. In Vitro Transcription of Viral RNA

To linearize the different plasmids, an XhoI restriction site downstream of the poly-A tail was used. Plasmids (10 µg) were linearized with 0.5 U/µL XhoI (Thermo Fisher, Waltham, MA, USA) in buffer R (Thermo Fisher, Waltham, MA, USA) for 2 h. The linearized DNA was purified with a 25:24:1 (*v*/*v*) mixture of phenol, chloroform, and isoamyl alcohol (Sigma-Aldrich, St. Louis, MO, USA), followed by a chloroform wash, ethanol sodium acetate precipitation, and additional washing with ethanol (75%). For in vitro RNA transcription, the mMESSAGE mMACHINE™ T7 transcription kit (Thermo Fisher, Waltham, MA, USA) was used according to the manufacturer’s instructions, with 1 µg linearized DNA and 1 µL GTP (30 mM). The in vitro transcribed RNA was purified with Microspin™ S-400 HR columns (Cytiva, Marlborough, MA, USA) and stored at −80 °C until further use.

### 2.5. Virus Rescue

In vitro transcribed RNA (2.5 µg) was transfected into BSR-T7 cells in six-well plates using a TransIT^®^-mRNA transfection kit (Mirus Bio, Madison, WI, USA) according to the manufacturer’s instructions (no RNA was used for mock transfection). After 8 h of incubation at 37 °C, the medium was removed, and the cells were washed with phosphate-buffered saline (PBS) before the addition of Opti-MEM™ I reduced-serum medium (Gibco, Carlsbad, CA, USA). After 72 h, the cell medium lysate (CML) or supernatant was collected, subjected to three freeze–thaw cycles, centrifuged at 2000× *g* for 5 min to remove the cell debris, and stored at −80 °C. For the infection experiments, the CML or supernatant of transfected BSR-T7 cells or infected CaCo-2 cells was treated with 10 µg/mL Trypsin IX (Sigma-Aldrich, St. Louis, MO, USA) for 45 min and incubated on CaCo-2 cells for 1 h. The supernatant was then removed, and the CaCo-2 cells were washed with PBS and further incubated in fresh Opti-MEM™ I reduced-serum medium (Gibco, Carlsbad, CA, USA) containing 0.2 µg/mL Trypsin IX (Sigma-Aldrich, St. Louis, MO, USA; Figure 1a). For virus titration, CaCo-2 cells were seeded in a 96-well plate and infected with 50 µL CML or supernatant in 10-fold serial dilutions. After 72 h, the cells were fixed and stained with mouse monoclonal antibody (mAb) 8E7 (400 ng/mL in 0.05% PBS-T) (Santa Cruz Biotechnology, Dallas, TX, USA), which was directed against the ORF2-encoded capsid protein of HAstV-1. Fifty percent of tissue culture infectious doses (TCID_50_s) were calculated using the Reed and Muench [58] method.

### 2.6. Luciferase Assay

BSR-T7 cells were transfected with 0.06 µg in vitro transcribed RNA per well in 96-well plates using the TransIT^®^-mRNA transfection kit (Mirus Bio, Madison, WI, USA) according to the manufacturer’s instructions (no RNA was used for mock transfection). After 2 h of incubation at 37 °C, the medium was removed, the cells were washed with PBS, and fresh medium was added.

Luciferase activity was measured using a Cytation™ 5 device (BioTek, Winooski, VT, USA) at 2, 8, 24, 48, and 72 h after transfection using the Nano-Glo^®^ Dual-Luciferase^®^ reporter assay system (Promega, Madison, WI, USA) according to the manufacturer’s instructions.

### 2.7. Cell Viability Assay

To assess cell viability, in vitro transcribed RNA was transfected into BSR-T7 cells (0.06 µg/well for the luminescence assay, 0.09 µg/well for virus rescue, and no RNA for mock), or CaCo-2 cells were infected in 96-well plates using the TransIT^®^-mRNA transfection kit (Mirus Bio, Madison, WI, USA). After 2 (luminescence assay) or 8 (virus rescue) hours of incubation at 37 °C, the medium was removed, the cells were washed with PBS, and fresh DMEM, high glucose, and pyruvate (Gibco, Carlsbad, CA, USA) or Opti-MEM™ I reduced-serum medium (Gibco, Carlsbad, CA, USA) were added. Absorption was measured using the Cytation™ 5 device (BioTek, Winooski, VT, USA) 72 h after transfection using the cell counting kit-8 (Sigma-Aldrich, St. Louis, MO, USA) according to the manufacturer’s instructions.

### 2.8. Immunofluorescence

BSR-T7 and CaCo-2 cells were fixed for 15 min in 4% (*w*/*v*) paraformaldehyde, followed by three washes with 0.05% (*v*/*v*) phosphate-buffered saline with Tween (PBS-T). The cells were then incubated with 0.5% (*v*/*v*) Triton X in 0.05% PBS-T for 20 min, followed by a blocking step with 10% (*v*/*v*) normal goat serum in 0.05% PBS-T for 20 min. The HAstV-1 capsid protein was detected using incubation with mAb 8E7 (400 ng/mL; Santa Cruz Biotechnology, Dallas, TX, USA) in 0.05% PBS-T for 2 h. Then, the cells were washed three times with 0.05% PBS-T and incubated with the goat antimouse immunoglobulin G (H+L) cross-adsorbed secondary antibody Alexa Fluor 555 (2 µg/mL in 0.05% PBS-T; Invitrogen, Carlsbad, CA, USA) and Thermo Scientific™ DAPI solution (1 µg/mL in 0.05% PBS-T; Thermo Fisher, Waltham, MA, USA) for 2 h. Finally, the cells were washed twice with PBS-T and once with distilled water. For microphotographs, the cells were seeded on coverslips and mounted on glass slides with Glycergel (Agilent, Santa Clara, CA, USA). Microphotographs were taken using an FV3000 confocal laser scanning microscope (Olympus, Shinjuku, Japan) and processed using Fiji [59] (ImageJ, open source).

### 2.9. In Situ Hybridization

To obtain further evidence of RNA replication, we performed in situ hybridization (ISH). Cells were seeded in an eight-well Nunc^®^ Lab-Tek^®^ Chamber Slide™ system (Merck KGaA, Darmstadt, Germany) and fixed with 10% neutral-buffered formalin 24 h after the transfection of 0.5 µg viral RNA (no RNA for mock). To detect the antigenomic RNA of HAstV-1, a customized RNA probe targeting the ORF1a region (nucleotide positions 86–1094; catalog No. 571951, ACDBio, Newark, CA, USA) was designed and used in combination with the RNAscope^®^ 2.5 HD Assay-RED (ACDBio, Newark, CA, USA) according to the manufacturer’s instructions. Microphotographs were taken using an Eclipse E600 microscope (Nikon, Minato, Japan) and an Axiocam 208 color camera (Zeiss, Jena, Germany). The images were processed using ZEN (blue edition; Zeiss, Jena, Germany) and PowerPoint365 (Microsoft, Redmond, WA, USA).

### 2.10. Statistical Analysis

Luciferase activity was compared using one-way analysis of variance (ANOVA) followed by Tukey’s multiple comparison test (*p* < 0.001). The TCID_50_ values were normalized to that of wt HAstV-1 and compared using one-way ANOVA. The analyses were performed using GraphPad Prism software (version 5.00 for Windows; GraphPad Software, San Diego, CA, USA).

## 3. Results

### 3.1. Human Astrovirus UTR Structures

**Figure 2:**
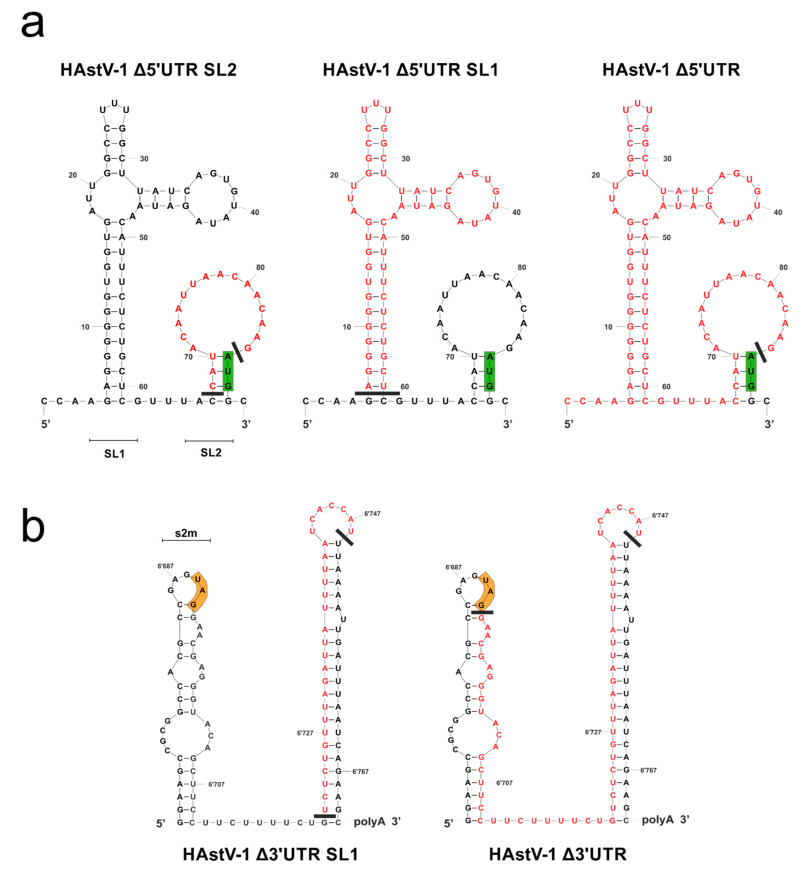
Secondary RNA structures predicted for the 5′ (**a**) and 3′ (**b**) UTRs of HAstV-1 and design of UTR deletion mutants. The deleted nucleotides are shown in red. The green boxes indicate the start codon of ORF1a, and the orange boxes indicate the stop codon of ORF2. These structure models were generated using mfold [51] and modified using CorelDraw X6 (version 16.0.0.707). SL, stem-loop; s2m, stem-loop 2 motif.

The 3′ UTRs of the classical HAstV strains were almost identical (87% to 100% sequence identity), but some differences were detected in the 5′ UTRs, especially in the nucleotides in proximity to the start codon of ORF1a (Figure S1). Overall, the UTRs of the classical HAstV strains appeared to be highly conserved, indicating their functional relevance. The mfold [51] and RNAfold [52,53] programs predicted two SL structures for the 5′ UTR (SL1 and SL2; Figure 2a) and the 3′ UTR (s2m and SL1; Figure 2b) of HAstV-1. In the 5′ UTR, the small SL upstream of the start codon (Δ5′ UTR SL2), the large SL at the 5′ terminus (Δ5′ UTR SL1), or the complete 5′ UTR (Δ5′ UTR) was deleted. In the 3′ UTR, part of the large SL upstream of the poly-A tail (Δ3′ UTR SL1) or almost the complete 3′ UTR (Δ3′ UTR) was deleted. The remaining nucleotides upstream of the poly-A tail were retained for use as a primer binding site for mutagenesis. The minimum free energy was −14.50 kcal/mol for the 5′ UTR and −26.90 kacal/mol for the 3′ UTR.

### 3.2. HAstV-1 5′ and 3′ UTR Deletion Mutants Are RNA-Replication-Competent

**Figure 3:**
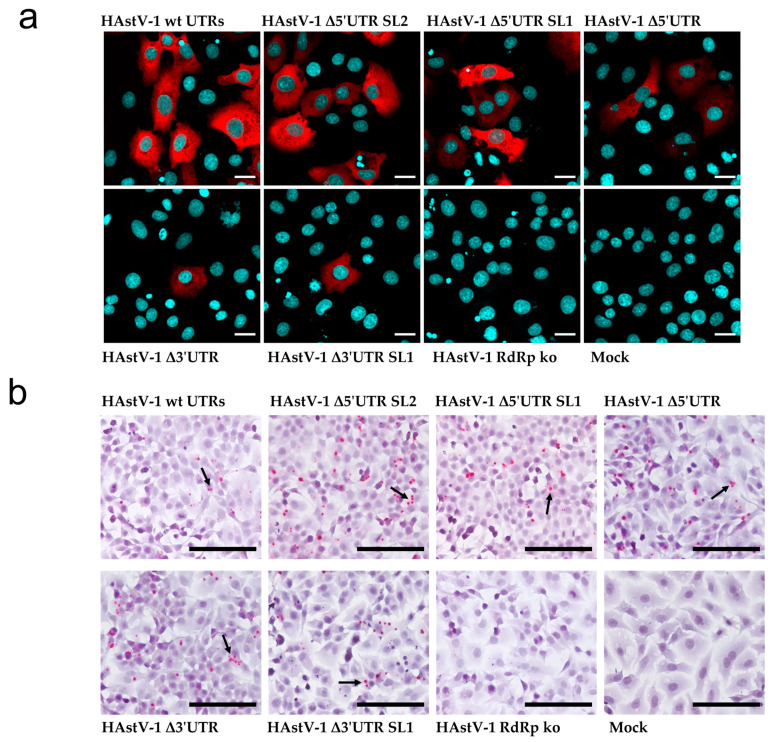
Translation of viral proteins and viral RNA replication in HAstV-1 UTR deletion mutants. (**a**) BSR-T7 cells transfected with wt HAstV-1 and deletion mutants. The HAstV capsid protein is labeled in red and cellular nuclei in blue. Scale bar = 20 µm. (**b**) Images from the in situ hybridization for antigenomic HAstV-1 RNA of BSR-T7 cells transfected with wt HAstV-1 and deletion mutants. Red dots and arrows indicate RNA probe hybridization. Scale bar = 50 µm, 40× magnification.

As expected, the capsid protein was readily detected in BSR-T7 cells transfected with wt RNA but not in those transfected with the RdRp ko mutant. The capsid protein was also expressed in the three 5′ UTR mutants, whereas positive staining was detected in few cells of the two 3′ UTR mutants (Figure 3a). As the capsid protein is translated from a subgenomic RNA, its expression provided indirect evidence of viral RNA replication. With ISH, we detected antigenomic RNA after transfection of the wt genome and all deletion mutants but not of the RdRp ko construct (Figure 3b). Taken together, these data support that all tested UTR deletion mutants are RNA-replication-competent, but viral protein expression in the 3′ UTR mutants seems to be impaired.

### 3.3. Viral Titers Are Decreased in HAstV-1 UTR Mutants

**Figure 4:**
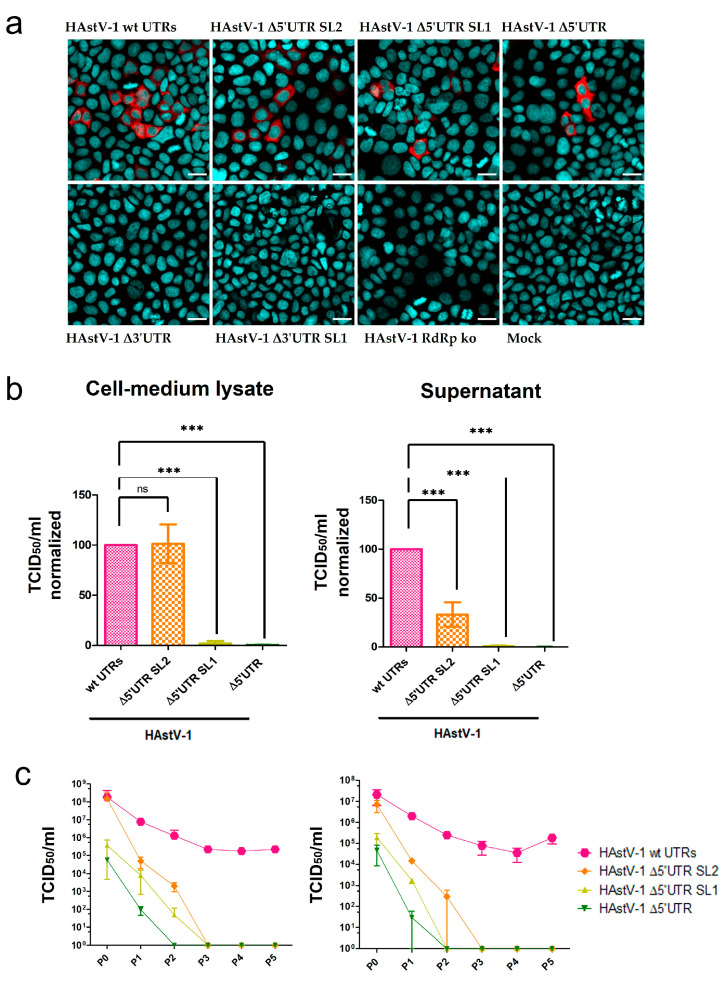
Compromised viral particle assembly and release of HAstV-1 UTR deletion mutants. (**a**) Immunofluorescence images of CaCo-2 cells infected with supernatants of transfected BSR-T7 samples. The HAstV-1 capsid protein is stained red, and nuclei are stained blue. Scale bar = 20 µm. (**b**) Titration of HAstV-1 5′ UTR mutants (after BSR-T7 cell transfection = P0) on CaCo-2 cells. The titers (quantified as the 50% tissue culture infectious dose (TCID_50_)/mL) were normalized to those of wt HAstV-1. Data for the 3′ UTR and HAstV-1 RdRp ko mutants are not shown because these mutants did not infect CaCo-2 cells. The error bars indicate standard deviations from three independent experiments. *** *p* < 0.001. (**c**) Virus titers of HAstV-1 mutants on CaCo-2 cells in sequential passages (*n* = 5). P0, after BSR-T7 cell transfection; P1–P5, after CaCo-2 cell infection (*n* = 2), ns = nonsignificant.

After the passage of the supernatants of transfected BSR-T7 cells to the CaCo-2 cells, all three 5′ UTR mutants were infectious (Figure 4a, upper panel), but the two 3′ UTR mutants were deficient in infectious virus production (Figure 4a, lower panel). The 5′ UTR mutants showed considerable differences in the number of infected cells. Virus titers of the Δ5′ UTR SL2 mutant were similar to those of wt HAstV-1 in the CML but were reduced by more than 50% in the supernatant (Figure 4b). The titers of the Δ5′ UTR SL1 and Δ5′ UTR mutants were strongly (~10^3^-fold) reduced compared with those of wt HAstV-1 in the CML and supernatant (Figure 4b). The titers of the 5′ UTR mutants and wt HAstV-1 decreased after the first passage. However, the titer of wt HAstV-1 remained stable over several passages, whereas those of all the 5′ UTR mutants were reduced to zero after a maximum of three CML and supernatant passages (Figure 4c). The cell viability assay revealed no negative effect of mutant or wt HAstV-1 replication (Figure S2). These results indicate that all investigated 5′ UTR mutants were replication-competent in principle but had compromised infectious particle assembly to the extent that they could not be passaged more than three times on CaCo-2 cells.

### 3.4. Different Protein Expression from Genomic amd Subgenomic RNAs in UTR Mutants

**Figure 5:**
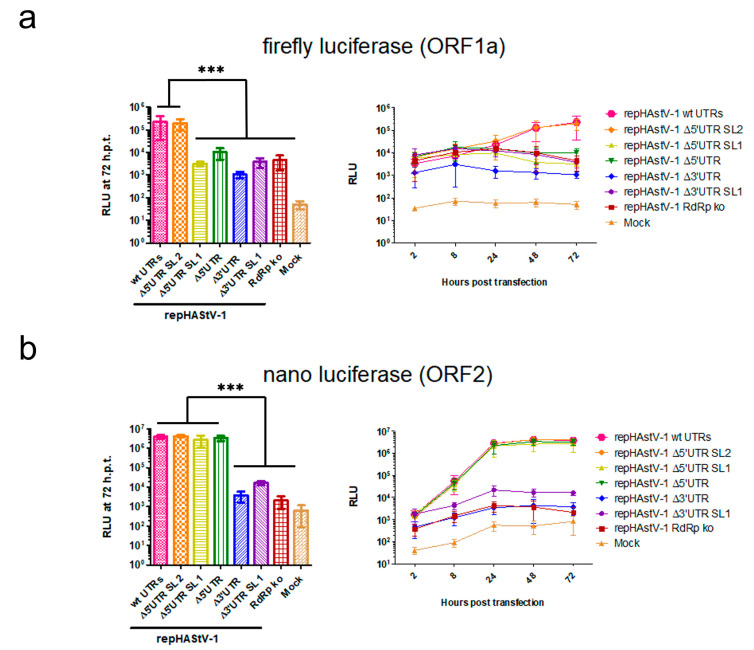
HAstV-1 UTR deletions compromise viral protein expression. Fluc (**a**) and nluc (**b**) activity at 72 h after transfection (hpt; left) and over time (2–72 h, right). *** *p* < 0.01. The experiments were conducted in triplicate. The error bars indicate standard deviations (*n* = 9). fluc, firefly luciferase; nluc, nanoluciferase; RLUs, relative luminescence units.

After transfection into BSR-T7 cells, fluc activity in ORF1a was decreased to levels similar to that in the RdRp ko mutant in all UTR mutants except repHAstV-1 Δ5′ UTR SL2 (Figure 5a). In contrast, nanoluciferase activity in ORF2 was decreased to levels similar to that in the RdRp ko mutant in the 3′ UTR mutants but was similar to that in the wt replicon in the 5′ UTR mutants (Figure 5b). Notably, cell viability did not differ significantly after transfection of the different replicon RNAs (Figure S2). Collectively, these data suggest that mutations in the 3′ and 5′ UTRs (except that involving only the 5′ UTR SL1) had a strong impact on the viral expression of nonstructural proteins. However, structural protein expression was only compromised by the 3′ UTR mutations.

## 4. Discussion

This study showed that the UTRs of HAstV-1, as of other viruses [31,33,35], are essential for its life cycle. HAstV-1 genomes with 3′ UTR deletions affecting SL1, alone and in combination with s2m, showed a deficient generation of infectious viral particles and viral protein translation from genomic and subgenomic RNA. However, ISH provided evidence of antigenomic RNA transcription in transfected cells (for viral RNA replication) at levels similar to that in wt HAstV-1. This finding indicates that nsp1a and nsp1ab expression occurred but at very low levels, possibly below the detection limit of our dual-reporter replicon system.

In contrast, the 5′ UTR mutants showed infectious virus generation. Upon the first passage in CaCo-2 cells, the viral titers were markedly reduced relative to the wt for the 5′ UTR SL1 and 5′ UTR deletion mutants. These mutants showed impaired protein translation from genomic but not subgenomic RNA, suggesting that 5′ UTR SL1 is functionally essential for the former. In contrast, titers for the Δ5′ UTR SL2 mutant virus were similar to that of the wt virus in CMLs and reduced by about half in cell culture supernatants. In the dual-reporter system, the 5′ UTR SL2 mutant replicon had expression levels similar to the wt replicons from genomic and subgenomic RNA. Thus, the lack of 5′ UTR SL2 may negatively affect viral particle release but not RNA replication or viral protein translation.

The observed reduction in titers for all of the 5′ UTR mutants in CML and the supernatant with passaging over time indicates that viral replication was compromised not only at the level of infectious particle release from cells but also at the level of viral particle assembly. This finding may indicate that the VPg or other proteins that are important for viral particle assembly did not bind correctly to the genomic RNA because an important RNA structure was disrupted. The immunodetection of the VPg in the supernatant of the transfected cells could clarify whether this protein remains linked to the genome when the 5′ UTR is partially or completely deleted. Our results could be biased by the presence of in vitro transcribed transfected RNA packaged during the first passages. This possibility would also explain the observation of an initial decrease in wt HAstV-1 titers over the first passages. Another explanation could be cell-dependent mechanisms, which do not allow the wt HAstV-1 to replicate to the higher titer. However, whereas the wt HAstV-1 UTRs efficiently form packable RNA, the mutant UTRs do not.

This study was limited by our inability to quantify and compare the levels of genomic and subgenomic RNA during RNA replication in the UTR mutants. Our attempts to achieve this using real-time quantitative polymerase chain reaction and Northern blotting yielded inconclusive results due to the large amounts of in vitro transcribed and transfected RNA species. Thus, we could not determine whether the differences in titer and luciferase activity resulted only from impaired protein translation or also from decreased RNA replication. Another point is that the length of the 5′ UTR may be very important for the viral life cycle. It would be interesting to have a construct of the same length as the wild-type 5′ UTR but with a different nucleotide sequence.

However, determining in which way the translation is affected by the mutations needs further investigation. It would be interesting to examine if the binding of proteins associated with translation initiation is disturbed in its binding capacities, as it is already known that PTB protein is crosslinked to the 3′ UTR of the HAstV-8 and when blocked, leads to reduced infectivity [46,47]. On the other hand, this finding reflects a difference from the 3′ UTR deletion mutants of picornaviruses, which continue to produce infectious viral particles [60], and that HAstV-1 can overcome mutations in the s2m but not in the rest of the 3′ UTR [50]. Another field to examine is whether similar host proteins are involved in astrovirus replication, such as those described for members of the *Caliciviridae* and *Picornaviridae* [28,61].

Further research is needed to gain a better understanding of the mechanisms underlying the findings presented here and the virus–host interactions. A major drawback affecting astrovirus research is the lack of knowledge about the exact processing of nonstructural and capsid proteins. Such knowledge would facilitate the development of an infectious reporter virus, which, in turn, could simplify the titration and quantification of proteins after infection. Moreover, the structures of these viruses could be studied in more detail using SHAPE-MAP [62], which enables the identification of nucleotides forming base pairs, as has been undertaken, for example, for the hepatitis C virus [63] and severe acute respiratory syndrome coronavirus 2 [64].

## 5. Conclusions

The results of this study indicate that the presence of HAstV-1 UTRs is essential for the viral life cycle, as 3′ UTR deletions almost completely abolish protein expression, and 5′ UTR deletions reduce infectious viral particle release. They lay a foundation for the further characterization of the roles of UTRs in astrovirus replication. Astrovirus UTRs may be interesting targets for antiviral development to provide new strategies for the treatment of intestinal and extraintestinal manifestations of astrovirus infection.

## Data Availability

Not applicable.

## Supplementary Materials

The following supporting information can be downloaded at https://www.mdpi.com/article/10.3390/v15061402/s1: Table S1: Primers used for the construction of human astrovirus 1 mutant viruses and dual-reporter replicons, Figure S1: Alignment of 5′ UTRs (A) and 3′ UTRs (B) of classical HAstV strains, Figure S2: Cell viability of transfected and infected cells [65,66].

## References

[1] Shi M., Lin X.-D., Chen X., Tian J.-H., Chen L.-J., Li K., Wang W., Eden J.-S., Shen J.-J., Liu L. (2018). The Evolutionary History of Vertebrate RNA Viruses. Nature.

[2] Bosch A., Pintó R.M., Guix S. (2014). Human Astroviruses. Clin. Microbiol. Rev..

[3] Bouzalas I.G., Wüthrich D., Walland J., Drögemüller C., Zurbriggen A., Vandevelde M., Oevermann A., Bruggmann R., Seuberlich T. (2014). Neurotropic Astrovirus in Cattle with Nonsuppurative Encephalitis in Europe. J. Clin. Microbiol..

[4] Li L., Diab S., McGraw S., Barr B., Traslavina R., Higgins R., Talbot T., Blanchard P., Rimoldi G., Fahsbender E. (2013). Divergent Astrovirus Associated with Neurologic Disease in Cattle. Emerg. Infect. Dis..

[5] Boujon C.L., Koch M.C., Kauer R.V., Keller-Gautschi E., Hierweger M.M., Hoby S., Seuberlich T. (2019). Novel Encephalomyelitis-Associated Astrovirus in a Muskox (*Ovibos Moschatus*): A Surprise from the Archives. Acta Vet. Scand..

[6] Küchler L., Rüfli I., Koch M.C., Hierweger M.M., Kauer R.V., Boujon C.L., Hilbe M., Oevermann A., Zanolari P., Seuberlich T. (2020). Astrovirus-Associated Polioencephalomyelitis in an Alpaca. Viruses.

[7] Pfaff F., Schlottau K., Scholes S., Courtenay A., Hoffmann B., Höper D., Beer M. (2017). A Novel Astrovirus Associated with Encephalitis and Ganglionitis in Domestic Sheep. Transbound. Emerg. Dis..

[8] Boros Á., Albert M., Pankovics P., Bíró H., Pesavento P.A., Phan T.G., Delwart E., Reuter G. (2017). Outbreaks of Neuroinvasive Astrovirus Associated with Encephalomyelitis, Weakness, and Paralysis among Weaned Pigs, Hungary. Emerg. Infect. Dis..

[9] Arruda B., Arruda P., Hensch M., Chen Q., Zheng Y., Yang C., Gatto I.R.H., Ferreyra F.M., Gauger P., Schwartz K. (2017). Porcine Astrovirus Type 3 in Central Nervous System of Swine with Polioencephalomyelitis. Emerg. Infect. Dis..

[10] Soares C.C., Maciel de Albuquerque M.C., Maranhão A.G., Rocha L.N., Ramírez M.L.G., Benati F.J., Timenetsky M.d.C., Santos N. (2008). Astrovirus Detection in Sporadic Cases of Diarrhea among Hospitalized and Non-Hospitalized Children in Rio De Janeiro, Brazil, from 1998 to 2004. J. Med. Virol..

[11] Castro C.J., Reynolds E., Monroe S.S., Marine R.L., Vinjé J. (2019). Complete Genome Sequences of Human Astrovirus Prototype Strains (Types 1 to 8). Microbiol. Resour. Announc..

[12] Cordey S., Brito F., Vu D.-L., Turin L., Kilowoko M., Kyungu E., Genton B., Zdobnov E.M., D’Acremont V., Kaiser L. (2016). Astrovirus VA1 Identified by Next-Generation Sequencing in a Nasopharyngeal Specimen of a Febrile Tanzanian Child with Acute Respiratory Disease of Unknown Etiology. Emerg. Microbes Infect..

[13] Cordey S., Vu D.L., Schibler M., L’Huillier A.G., Brito F., Docquier M., Posfay-Barbe K.M., Petty T.J., Turin L., Zdobnov E.M. (2016). Astrovirus MLB2, a New Gastroenteric Virus Associated with Meningitis and Disseminated Infection. Emerg. Infect. Dis..

[14] Schibler M., Brito F., Zanella M.C., Zdobnov E.M., Laubscher F., L’Huillier A.G., Ambrosioni J., Wagner N., Posfay-Barbe K.M., Docquier M. (2019). Viral Sequences Detection by High-Throughput Sequencing in Cerebrospinal Fluid of Individuals with and without Central Nervous System Disease. Genes.

[15] Vu D.-L., Cordey S., Brito F., Kaiser L. (2016). Novel Human Astroviruses: Novel Human Diseases?. J. Clin. Virol..

[16] Vu D.-L., Bosch A., Pintó R.M., Ribes E., Guix S. (2019). Human Astrovirus MLB Replication In Vitro: Persistence in Extraintestinal Cell Lines. J. Virol..

[17] Vu D.-L., Bosch A., Pintó R.M., Guix S. (2017). Epidemiology of Classic and Novel Human Astrovirus: Gastroenteritis and Beyond. Viruses.

[18] Wylie K.M., Mihindukulasuriya K.A., Sodergren E., Weinstock G.M., Storch G.A. (2012). Sequence Analysis of the Human Virome in Febrile and Afebrile Children. PLoS ONE.

[19] Holtz L.R., Bauer I.K., Rajendran P., Kang G., Wang D. (2011). Astrovirus MLB1 Is Not Associated with Diarrhea in a Cohort of Indian Children. PLoS ONE.

[20] Holtz L.R., Wylie K.M., Sodergren E., Jiang Y., Franz C.J., Weinstock G.M., Storch G.A., Wang D. (2011). Astrovirus MLB2 Viremia in Febrile Child. Emerg. Infect. Dis..

[21] Janowski A.B. (2021). Beyond the Gastrointestinal Tract: The Emerging and Diverse Tissue Tropisms of Astroviruses. Viruses.

[22] Willcocks M.M., Brown T.D.K., Madeley C.R., Carter M.J.Y. (1994). The Complete Sequence of a Human Astrovirus. J. Gen. Virol..

[23] Lulla V., Firth A.E. (2020). A Hidden Gene in Astroviruses Encodes a Viroporin. Nat. Commun..

[24] Fuentes C., Bosch A., Pintó R.M., Guix S. (2012). Identification of Human Astrovirus Genome-Linked Protein (VPg) Essential for Virus Infectivity. J. Virol..

[25] Monroe S.S., Stine S.E., Gorelkin L., Herrmann J.E., Blacklow N.R., Glass R.I. (1991). Temporal Synthesis of Proteins and RNAs during Human Astrovirus Infection of Cultured Cells. J. Virol..

[26] Willcocks M.M., Carter M.J. (1993). Identification and Sequence Determination of the Capsid Protein Gene of Human Astrovirus Serotype 1. FEMS Microbiol. Lett..

[27] Sorokin I.I., Vassilenko K.S., Terenin I.M., Kalinina N.O., Agol V.I., Dmitriev S.E. (2021). Non-Canonical Translation Initiation Mechanisms Employed by Eukaryotic Viral MRNAs. Biochemistry.

[28] Alhatlani B., Vashist S., Goodfellow I. (2015). Functions of the 5′ and 3′ Ends of Calicivirus Genomes. Virus Res..

[29] Dreher T.W. (1999). Functions of the 3′-Untranslated Regions of Positive Strand Rna Viral Genomes. Annu. Rev. Phytopathol..

[30] Rasekhian M., Roohvand F., Habtemariam S., Marzbany M., Kazemimanesh M. (2021). The Role of 3′UTR of RNA Viruses on MRNA Stability and Translation Enhancement. Mini Rev. Med. Chem..

[31] Jackson R.J., Hunt S.L., Gibbs C.L., Kaminski A. (1994). Internal Initiation of Translation of Picornavirus RNAs. Mol. Biol. Rep..

[32] Jang S.K., Kräusslich H.G., Nicklin M.J., Duke G.M., Palmenberg A.C., Wimmer E. (1988). A Segment of the 5′ Nontranslated Region of Encephalomyocarditis Virus RNA Directs Internal Entry of Ribosomes during in Vitro Translation. J. Virol..

[33] Pelletier J., Sonenberg N. (1988). Internal Initiation of Translation of Eukaryotic MRNA Directed by a Sequence Derived from Poliovirus RNA. Nature.

[34] Perera R., Daijogo S., Walter B.L., Nguyen J.H.C., Semler B.L. (2007). Cellular Protein Modification by Poliovirus: The Two Faces of Poly(RC)-Binding Protein. J. Virol..

[35] Zoll J., Heus H.A., van Kuppeveld F.J.M., Melchers W.J.G. (2009). The Structure–Function Relationship of the Enterovirus 3′-UTR. Virus Res..

[36] Bertolotti-Ciarlet A., Crawford S.E., Hutson A.M., Estes M.K. (2003). The 3′ End of Norwalk Virus MRNA Contains Determinants That Regulate the Expression and Stability of the Viral Capsid Protein VP1: A Novel Function for the VP2 Protein. J. Virol..

[37] Blyn L.B., Towner J.S., Semler B.L., Ehrenfeld E. (1997). Requirement of Poly(RC) Binding Protein 2 for Translation of Poliovirus RNA. J. Virol..

[38] Vashist S., Urena L., Chaudhry Y., Goodfellow I. (2012). Identification of RNA-Protein Interaction Networks Involved in the Norovirus Life Cycle. J. Virol..

[39] Gutiérrez-Escolano A.L., Vázquez-Ochoa M., Escobar-Herrera J., Hernández-Acosta J. (2003). La, PTB, and PAB Proteins Bind to the 3′ Untranslated Region of Norwalk Virus Genomic RNA. Biochem. Biophys. Res. Commun..

[40] Gutiérrez-Escolano A.L., Brito Z.U., del Angel R.M., Jiang X. (2000). Interaction of Cellular Proteins with the 5′ End of Norwalk Virus Genomic RNA. J. Virol..

[41] Cancio-Lonches C., Yocupicio-Monroy M., Sandoval-Jaime C., Galvan-Mendoza I., Ureña L., Vashist S., Goodfellow I., Salas-Benito J., Gutiérrez-Escolano A.L. (2011). Nucleolin Interacts with the Feline Calicivirus 3′ Untranslated Region and the Protease-Polymerase NS6 and NS7 Proteins, Playing a Role in Virus Replication. J. Virol..

[42] López-Manríquez E., Vashist S., Ureña L., Goodfellow I., Chavez P., Mora-Heredia J.E., Cancio-Lonches C., Garrido E., Gutiérrez-Escolano A.L. (2013). Norovirus Genome Circularization and Efficient Replication Are Facilitated by Binding of PCBP2 and HnRNP A1. J. Virol..

[43] Kim Y.K., Jang S.K. (1999). La Protein Is Required for Efficient Translation Driven by Encephalomyocarditis Virus Internal Ribosomal Entry Site. J. Gen. Virol..

[44] Svitkin Y.V., Meerovitch K., Lee H.S., Dholakia J.N., Kenan D.J., Agol V.I., Sonenberg N. (1994). Internal Translation Initiation on Poliovirus RNA: Further Characterization of La Function in Poliovirus Translation in Vitro. J. Virol..

[45] Witherell G.W., Gil A., Wimmer E. (1993). Interaction of Polypyrimidine Tract Binding Protein with the Encephalomyocarditis Virus MRNA Internal Ribosomal Entry Site. Biochemistry.

[46] Espinosa-Hernández W., Velez-Uriza D., Valdés J., Vélez-Del Valle C., Salas-Benito J., Martínez-Contreras R., García-Espítia M., Salas-Benito M., Vega-Almeida T., De Nova-Ocampo M. (2014). PTB Binds to the 3′ Untranslated Region of the Human Astrovirus Type 8: A Possible Role in Viral Replication. PLoS ONE.

[47] De Nova-Ocampo M., Soliman M.C., Espinosa-Hernández W., Velez-Del Valle C., Salas-Benito J., Valdés-Flores J., García-Morales L. (2019). Human Astroviruses: In Silico Analysis of the Untranslated Region and Putative Binding Sites of Cellular Proteins. Mol. Biol. Rep..

[48] Robertson M.P., Igel H., Baertsch R., Haussler D., Ares M., Scott W.G. (2005). The Structure of a Rigorously Conserved RNA Element within the SARS Virus Genome. PLoS Biol..

[49] Lulla V., Wandel M.P., Bandyra K.J., Ulferts R., Wu M., Dendooven T., Yang X., Doyle N., Oerum S., Beale R. (2021). Targeting the Conserved Stem Loop 2 Motif in the SARS-CoV-2 Genome. J. Virol..

[50] Keep S., Dowgier G., Lulla V., Britton P., Oade M., Freimanis G., Tennakoon C., Jonassen C.M., Tengs T., Bickerton E. (2023). Deletion of the S2m RNA Structure in the Avian Coronavirus Infectious Bronchitis Virus and Human Astrovirus Results in Sequence Insertions. J. Virol..

[51] Zuker M. (2003). Mfold Web Server for Nucleic Acid Folding and Hybridization Prediction. Nucleic Acids Res..

[52] Gruber A.R., Lorenz R., Bernhart S.H., Neuböck R., Hofacker I.L. (2008). The Vienna RNA Websuite. Nucleic Acids Res..

[53] Lorenz R., Bernhart S.H., Höner zu Siederdissen C., Tafer H., Flamm C., Stadler P.F., Hofacker I.L. (2011). ViennaRNA Package 2.0. Algorithms Mol. Biol..

[54] Geigenmüller U., Ginzton N.H., Matsui S.M. (1997). Construction of a Genome-Length CDNA Clone for Human Astrovirus Serotype 1 and Synthesis of Infectious RNA Transcripts. J. Virol..

[55] Vázquez A.L., Alonso J.M.M., Parra F. (2000). Mutation Analysis of the GDD Sequence Motif of a Calicivirus RNA-Dependent RNA Polymerase. J. Virol..

[56] Szymczak-Workman A.L., Vignali K.M., Vignali D.A.A. (2012). Design and Construction of 2A Peptide-Linked Multicistronic Vectors. Cold Spring Harb. Protoc..

[57] Buchholz U.J., Finke S., Conzelmann K.-K. (1999). Generation of Bovine Respiratory Syncytial Virus (BRSV) from CDNA: BRSV NS2 Is Not Essential for Virus Replication in Tissue Culture, and the Human RSV Leader Region Acts as a Functional BRSV Genome Promoter. J. Virol..

[58] Reed L.J., Muench H. (1938). A simple method of estimating fifty per cent endpoints. Am. J. Epidemiol..

[59] Schindelin J., Arganda-Carreras I., Frise E., Kaynig V., Longair M., Pietzsch T., Preibisch S., Rueden C., Saalfeld S., Schmid B. (2012). Fiji: An Open-Source Platform for Biological-Image Analysis. Nat. Methods.

[60] Todd S., Towner J.S., Brown D.M., Semler B.L. (1997). Replication-Competent Picornaviruses with Complete Genomic RNA 3′ Noncoding Region Deletions. J. Virol..

[61] Martínez-Salas E., Francisco-Velilla R., Fernandez-Chamorro J., Lozano G., Diaz-Toledano R. (2015). Picornavirus IRES Elements: RNA Structure and Host Protein Interactions. Virus Res..

[62] Siegfried N.A., Busan S., Rice G.M., Nelson J.A.E., Weeks K.M. (2014). RNA Motif Discovery by SHAPE and Mutational Profiling (SHAPE-MaP). Nat. Methods.

[63] Mauger D.M., Golden M., Yamane D., Williford S., Lemon S.M., Martin D.P., Weeks K.M. (2015). Functionally Conserved Architecture of Hepatitis C Virus RNA Genomes. Proc. Natl. Acad. Sci. USA.

[64] Huston N.C., Wan H., Strine M.S., de Cesaris Araujo Tavares R., Wilen C.B., Pyle A.M. (2021). Comprehensive in Vivo Secondary Structure of the SARS-CoV-2 Genome Reveals Novel Regulatory Motifs and Mechanisms. Mol. Cell.

[65] Thompson J.D., Higgins D.G., Gibson T.J. (1994). CLUSTAL W: Improving the sensitivity of progressive multiple sequence alignment through sequence weighting, position-specific gap penalties and weight matrix choice. Nucleic Acids Res..

[66] Tamura K., Stecher G., Kumar S. (2021). MEGA11: Molecular Evolutionary Genetics Analysis version 11. Mol. Biol. Evol..

