# Supplementary material for: The Roles of the 5′ and 3′ Untranslated Regions in Human Astrovirus Replication
Source: Viruses. 2023 Jun 20;15(6):1402. doi: 10.3390/v15061402 (PMC10302652; doi:10.3390/v15061402)
Supplement: Supplementary file 1 [file viruses-15-01402-s001.zip › viruses-2404800-supplementary.pdf]

**Table S1**: Primers used for the construction of Human Astrovirus 1 mutant viruses and dual-reporter replicons

| | |
|---|---|
| HA1_del_5'UTR_SL2_F | CTCGTTTAATGGCACACGGTGAGCCA |
| HA1_del_5'UTR_SL2_R | GTGCCATTAAACGAGCAGAGAAATGTTATCT |
| HA1_del_5'UTR_SL1_F | ATAGCCAAGTTTACCATACAATTAACAACAAGA |
| HA1_del_5'UTR_SL1_R | GGTAAACTTGGCTATAGTGAGTCGTATTAGGT |
| HA1_del_5'UTR_F | CACTATAGATGGCACACGGTGAGCCA |
| HA1_del_5'UTR_R | GTGCCATCTATAGTGAGTCGTATTAGGTATT |
| HA1_del_3'UTR_SL1_F | TCTTTTCTTTGATTTAATCAGAAGCAAAAAAAA |
| HA1_del_3'UTR__SL1_F | AAATCAAAGAAAAGAAGGAAGCTGTACC |
| HA1_del_3'UTR_F | CCGAGTAGTTGATTTAATCAGAAGC |
| HA1_del_3'UTR_R | AATCAACTACTCGGCGTGGCCGCG |
| ins_fluc_ORF1_HA1_F | ACACGGTGAGATGGAAGACGCCAAAAACATAAAG |
| ins_fluc_ORF1_HA1_R | TATGGCTCACCGTGTGCCATTTACAATTTGGACTTTCCGCCC |
| lin_HA1_ORF1_15bp_F | ATGGCACACGGTGAGCCATAC |
| lin_HA1_ORF1_15bp_R | CGTCTTCCATCTCACCGTGTGCCATCTTGTTGTTAATTGTATGGTAAACG |
| lin_2A_ORF1_HA1_F | ATGGCACACGGTGAGCCA |
| lin_2A_ORF1_HA1_R | CGTCTTCCATCTCACCGTGTGCCATCTTGTTGTTAATTGTATGGTAAACG |
| ins_2A_ORF1_HA1_F | ACACGGTGAGATGGAAGACGCCAAAAACATAAAG |
| Ins_2A_ORF1_HA1_R | CTCACCGTGTGCCATAGGGCCGGGATTCTCTTCCACGTCACCAGCCTGCTTCAGGAGGCTGAAGTTGGTGGCTCCGG ACCCTTACAATTTGGACTTTCCGCCCT |
| HA1_del STOP_flucO2l_F | CCAAATTGGGGTCCGGAGCCACCAAC |
| HA1_del STOP_flucO2l_R | CGGACCCCAATTTGGACTTTCCGCCCTTCTTG |
| HA1_ORF1_45bp ins_F | GGTCGTGAAGCGATTCACAGATGTCTGCCTGTTCATCC |
| HA1_ORF1_45bp ins_R | TTTGGCGTCTTCCATATCTTTGTCAGGTTTAGAGC |
| lin_pAVIC_45bp_F | ATGGAAGACGCCAAAAACATAAAG |
| lin_pAVIC_45bp_R | AATCGCTTCACGACCACG |
| Lin_ORF2_GFP/nluc_F | AAATCAGTCAAGATTACAGTCAATT |
| Lin_ORF2_GFP/nluc_R | ATCTCGGCCCCTAGATTGT |
| Ins_GFP/nluc_F | TCTAGGGGCCGAGATATGGTGTCCAAGGGCGAAGAGC |
| Ins_GFP/nluc_R | AATCTTGACTGATTTTCACGCCAGAATGCGTTCG |
| HA1_GDG_Muta_F | GGAGATGGGAGGCTTTCTACAACACCTTCG |
| HA1_GDG_Muta_R | AAGCCTCCCATCTCCATAAACTACAGTGTCAT |

**a)** 5′UTR

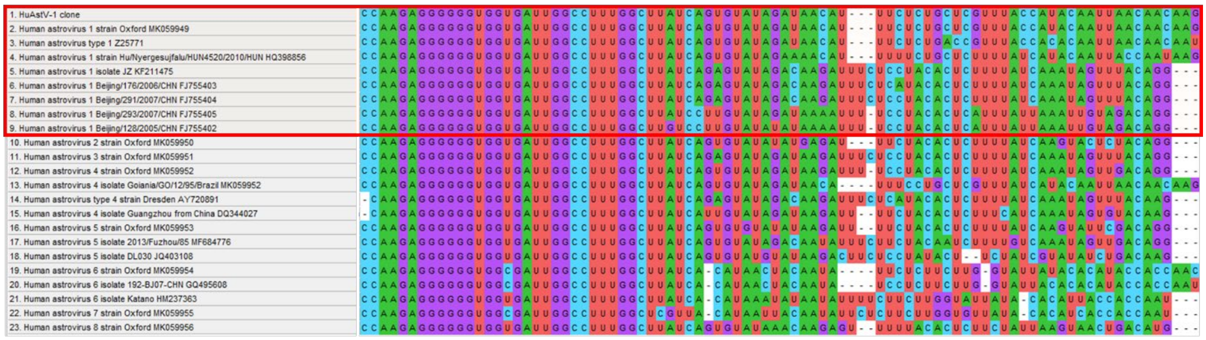


**b)** 3′UTR

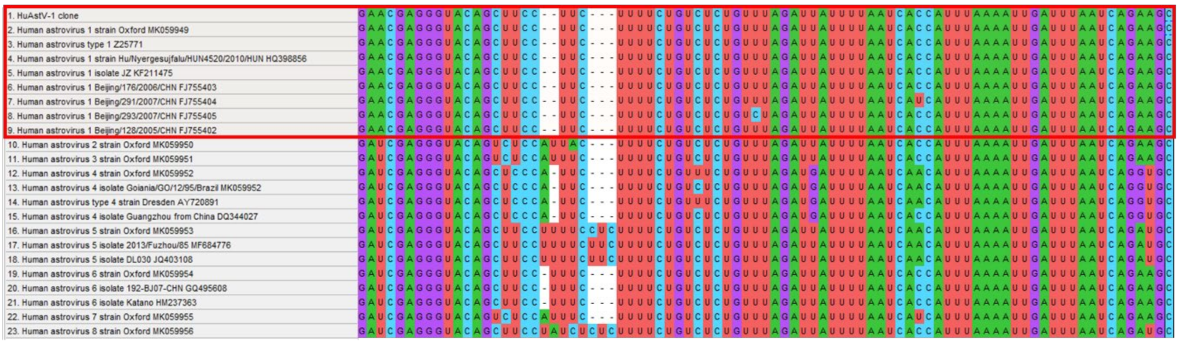


**Figure S1**: Alignment of 5′UTRs (A) and 3′UTR (B) of classical HuAstV strains. The sequences of the HAstV-1 are indicated by the red square. The alignment was performed with ClustalW [1] and the visualization with Mega X [2].

References:

[1] Thompson JD, Higgins DG, Gibson TJ (1994). CLUSTAL W: improving the sensitivity of progressive multiple sequence alignment through sequence weighting, position-specific gap penalties and weight matrix choice. Nucleic Acids Res. 22:4673-80.

[2] Tamura K, Stecher G, Kumar S. (2021) MEGA11: Molecular Evolutionary Genetics Analysis version 11. Molecular Biology and Evolution 38:3022-3027

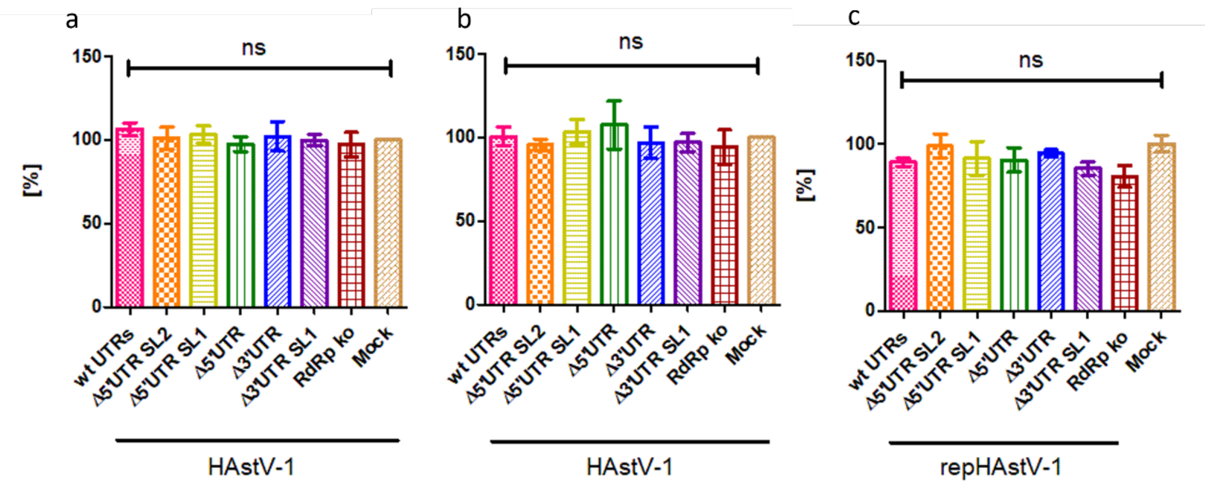


**Figure S2**: Cell viability of transfected and infected cells. (a) Transfected BSR-T7 with HuAstV-1 clone and mutants. (b) Infected CaCo-2 cells with HuAstV-1 clone and mutants. (c) Transfected BSR-T7 with HuAstV-1 replicons. All measurement were normalized to the corresponding mock. All experiments were performed trice. For statistical analysis a one-way analysis of variance (ANOVA) followed by a Tukey's multiple comparison test was performed using GraphPad Prism version 5.00 for Windows, GraphPad Software, San Diego California USA.
